# Multiple brain networks contribute to the acquisition of bias in perceptual decision-making

**DOI:** 10.3389/fnins.2015.00063

**Published:** 2015-03-05

**Authors:** Mei-Yen Chen, Koji Jimura, Corey N. White, W. Todd Maddox, Russell A. Poldrack

**Affiliations:** ^1^Department of Psychology, The University of Texas at AustinAustin, TX, USA; ^2^Precision and Intelligence Laboratory, Tokyo Institute of TechnologyTokyo, Japan; ^3^Department of Psychology, Stanford UniversityStanford, CA, USA

**Keywords:** decision-making, fMRI, motion discrimination, reinforcement learning, reward

## Abstract

Bias occurs in perceptual decisions when the reward associated with a particular response dominates the sensory evidence in support of a choice. However, it remains unclear how this bias is acquired and once acquired, how it influences perceptual decision processes in the brain. We addressed these questions using model-based neuroimaging in a motion discrimination paradigm where contextual cues suggested which one of two options would receive higher rewards on each trial. We found that participants gradually learned to choose the higher-rewarded option in each context when making a perceptual decision. The amount of bias on each trial was fit well by a reinforcement-learning model that estimated the subjective value of each option within the current context. The brain mechanisms underlying this bias acquisition process were similar to those observed in reward-based decision tasks: prediction errors correlated with the fMRI signals in ventral striatum, dlPFC, and parietal cortex, whereas the amount of acquired bias correlated with activity in ventromedial prefrontal (vmPFC), dorsolateral frontal (dlPFC), and parietal cortices. Moreover, psychophysiological interaction analysis revealed that as bias increased, functional connectivity increased within multiple brain networks (dlPFC-vmPFC-visual, vmPFC-motor, and parietal-anterior-cingulate), suggesting that multiple mechanisms contribute to bias in perceptual decisions through integration of value processing with action, sensory, and control systems. These provide a novel link between the neural mechanisms underlying perceptual and economic decision-making.

## Introduction

Decisions are driven both by the objective evidence presented to an individual and by the outcomes that the individual has learned to expect from the past. For decades, research in the neural mechanisms that process each of the two factors in decision-making has proceeded in parallel with little crosstalk. The literature on perceptual decisions has focused on how an individual's choices are influenced by the quality of sensory evidence, while the literature on economic decisions has emphasized how an individual's choices are driven by the expected reward learned from previous choice outcomes (Montague and Berns, 2002; Glimcher, 2011; Lee et al., 2012). Recently, converging evidence from animal physiology (Ding and Gold, 2013) and human neuroimaging (Summerfield and Tsetsos, 2012) has motivated a call to investigate behavior that links these two factors in order to identify the domain general neural mechanisms of decision-making processes. In this vein, here we report a study that examines the neural mechanisms underlying a well-known phenomenon—reward-induced bias in perceptual decisions (Edwards, 1965; Green and Swets, 1966; Maddox, 2002).

When prompted to classify sensory information as one of the two alternatives leading to asymmetric payoffs, both humans and animals tend to prefer the higher-rewarded alternative even though the sensory information may suggest the lower-rewarded alternative (Liston and Stone, 2008; Whiteley and Sahani, 2008; Feng et al., 2009; Fleming et al., 2010; Rorie et al., 2010; Summerfield and Koechlin, 2010; Mulder et al., 2012). In behavior studies, this choice preference can be identified from the sigmoidal relationship between the strength of sensory evidence and the probability of choosing one of the alternatives, or psychometric function. The horizontal shift of the indecision point in the psychometric function indicates the amount of choice preference, or bias, in perceptual decisions (Green and Swets, 1966; Macmillan and Creelman, 2004; Gold and Ding, 2013). The choice preference also can be characterized by reaction time since these biased choices usually are made faster (Summerfield and Koechlin, 2010; Mulder et al., 2012). Taken together, the drift-diffusion model analysis (Ratcliff, 1978) of the choices and reaction time suggest that information about reward may influence the starting point for individuals to accumulate sensory evidence in perceptual decisions (Summerfield and Koechlin, 2010; Mulder et al., 2012), such that less sensory evidence is required in order to support the more beneficial option.

Although the phenomenon and theoretical work are well documented at the behavioral level, the neural mechanisms through which reward information influences perceptual decisions remain an open question. One possible mechanism is that reward representations result in top-down influences on perceptual and motor regions, similar to the effects of a task set (Summerfield et al., 2006; Yeung et al., 2006) which selectively facilitates the sensory and motor systems to favor the option with higher-reward. If this account were valid, one would expect to observe two results. First, the activation of frontal and parietal cortices should positively correlate with the amount of reward-induced bias in perceptual decisions. Second, functional connectivity between frontal-parietal cortices and sensory-motor cortices should also increase when individuals express bias in perceptual decisions. However, to the best of our knowledge, only the first part of the theory has been supported in the literature (Fleming et al., 2010; Summerfield and Koechlin, 2010; Mulder et al., 2012); the second part of the theory remains untested.

Another potential mechanism for the development of bias is a reinforcement learning mechanism implemented in the dopaminergic system (Schultz, 1998; Sutton and Barto, 1998; Nomoto et al., 2010; Lee et al., 2012) through which information about reward can be transformed into subjective value and influence the perceptual decision making process (Rao, 2010; Bogacz and Larsan, 2011). This learned value may drive action selection (Wunderlich et al., 2009) and dominate sensory process in perceptual decisions. Nevertheless, until now, no empirical study has directly examined the involvement of reinforcement learning signals during bias acquisition process and the integration of subjective value into the neural mechanisms for perceptual decisions.

In the present study, we examined how bias is developed in perceptual decision-making and once developed how it influences the perceptual decision-making process in the brain. During fMRI acquisition, participants performed a motion discrimination task (Newsome et al., 1989; Britten et al., 1993; Shadlen and Newsome, 2001) with pre-trial cues signaling one of two different reward contexts. Trial-wise reward feedback was delivered to participants so that a correct response to one of the motion directions was reinforced more strongly in each reward context. To maximize reward, the participant must combine information about the stimulus and the potential reward, such that predicted reward exerts a greater effect on choices when the stimulus is weak. As the experiment proceeded, the indecision point of perceptual choices gradually shifted when it was measured in different reward contexts. This shift was quantified using a reinforcement-learning model that estimated the subjective value of each option according to the association of reward prediction error (RPE) and the reward contexts (Watkins and Dayan, 1992). Consistent with a role for value learning mechanisms, the RPE signals associated with the acquisition of the bias in perceptual decisions positively correlated with the activation of ventral striatum, dlPFC, and parietal cortex. As this bias developed, functional connectivity patterns suggested that the value signals that contributed to bias integrate into multiple networks involved in motor preparation (vmPFC-motor cortex), stimulus evaluation (frontal-vmPFC-visual cortex), and cognitive control (parietal-anterior cingulate cortex [ACC]) during perceptual decision-making. These results enhance our understanding of the neural mechanisms underlying bias acquisition at the computational level, and provide a fundamental linkage between perceptual and economic decision-making processes in the brain.

## Materials and methods

### Participants

Twenty-four human participants completed the behavioral paradigm in the MRI scanner (12 females, 12 males; age range: 18–30). One participant was excluded because of extreme parameter estimates (with orders of magnitude different from the other subjects, which may be caused by excessive noise or head movement) in the fMRI data analysis to ensure against violations of normality in the group analysis. All participants were recruited through posted flyers and were prescreened. They were free of any self-reported neurological or psychiatric diseases, had normal or corrected-to-normal visual acuity and normal color vision, and right-handed. They gave written informed consent for participation. The Institutional Review Board of the University of Texas at Austin approved all experimental procedures.

### Stimuli

All stimuli were generated in Matlab version 7.10.0, using the Psychophysics Toolbox extension, version 3.0.10 (Brainard, 1997; Pelli, 1997). Each motion stimulus was composed of 150 white dots moving inside a donut-shaped display patch on a black background. The display patch was centered on the screen and extended from 4 to 8° of visual-angle. Within the display patch, every dot moved at the speed of 8° of visual-angle per second. Some dots moved coherently toward one direction while the others moved randomly. The percentage of coherently moving dots determined the motion strength (coherence level). The presentation of the dots was controlled to remove local motion signals following the standard method for generating the motion stimuli (Newsome et al., 1989; Britten et al., 1993; Shadlen and Newsome, 2001; Palmer et al., 2005). That is, upon stimulus onset, the dots were presented at new random locations on each of the first three frames. They were relocated after two subsequent frames, so that the dots in frame 1 were repositioned in frame 4, and the dots in frame 2 were repositioned in frame 5, etc. When repositioned, each dot was either randomly presented at the new location or aligned with the pre-determined motion direction (upward or downward), depending on the pre-determined motion strength on that trial. Each stimulus was composed of 12 video frames with 60 Hz video frame refresh rates.

### Procedures and task

Participants first performed a practice session in the laboratory to familiarize themselves with the random-dot motion discrimination task. In the practice session, a trial began with a red fixation cross that was presented at the center of the display screen for 1.5 s. Then, a patch of moving dots was presented for 200 ms. After the stimulus offset, participants had to decide whether the global motion direction of these dots was upward or downward by pressing the corresponding spatially congruent buttons within 600 ms. Error feedback was presented for 1.5 s for incorrect or slow responses; otherwise, the next trial continued immediately with presentation of the fixation cross. On each trial, the motion stimulus was a random sample from one of the 9 coherence levels (0, ±6, ±12, ±64, ±80%; positive sign: upward motion, negative sign: downward motion). The correct response for 0% coherence trials was decided using a random number generator, so that the probability of being either correct or incorrect on this trial type was equal over the entire experiment. The total of 540 trials (9 coherence levels × 60 repetition) was broken down into six 90-trial blocks. The participants could take a break after completing each block.

**Figure 1:**
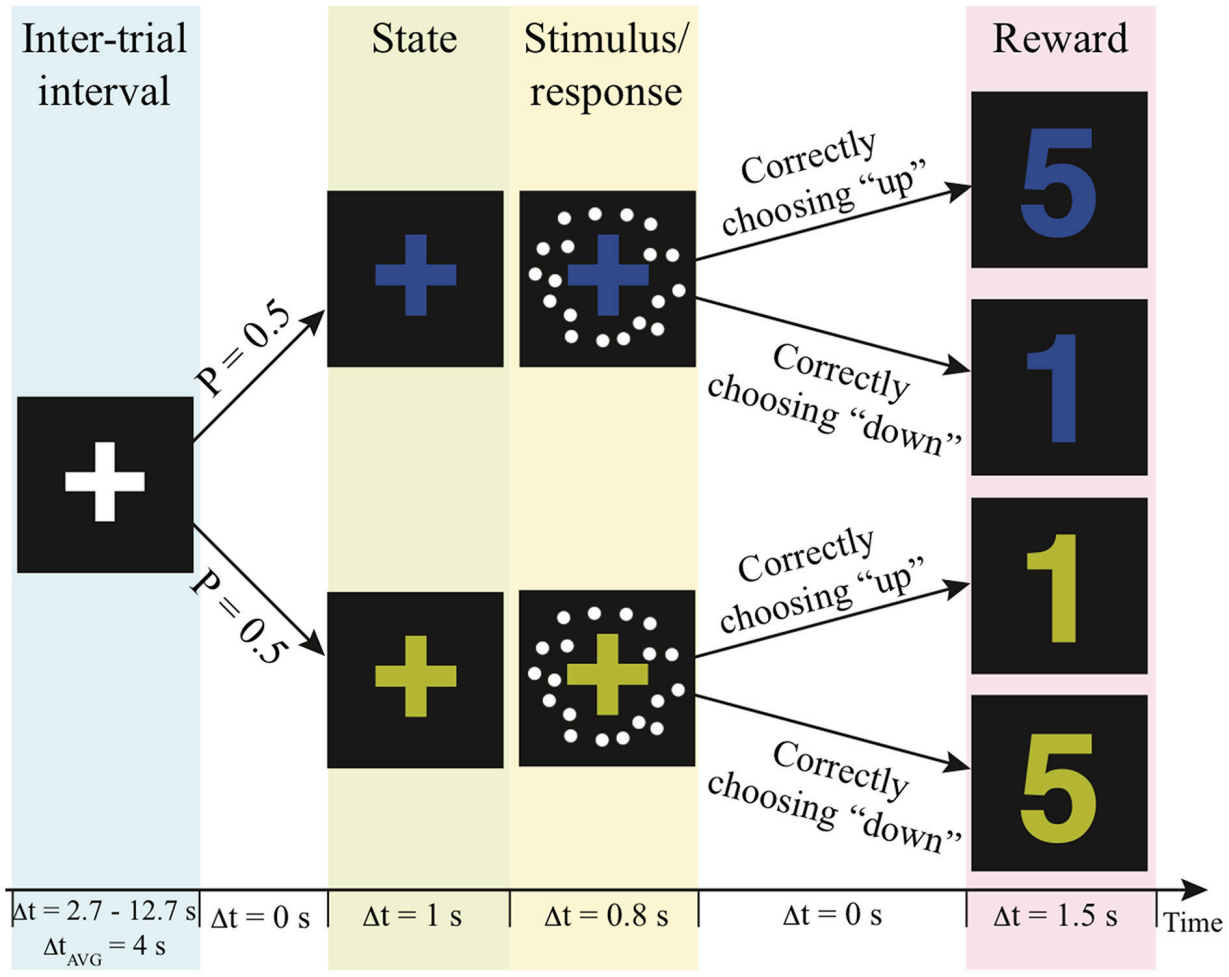
**Experimental paradigm**. Each trial is composed of a context, motion stimulus (illustrated as white dots), and reward points. The number of reward points that one can earn depends on the context and the choice. P, probability; Δt, duration; AVG, average; s, second.

The fMRI scan was conducted no more than 7 days after the practice session. In the scanner, participants were asked to decide the motion direction of moving-dots presented in two independent reward contexts with a goal to get as many reward points as possible over the experiment. The task structure and the timeline of events on a trial are illustrated in Figure 1. Before a trial started, a white fixation-cross presented at the center of the display screen during the jittered inter-trial interval (truncated exponential distribution; mean: 4 s, range 2.7–12.7 s). At the beginning of a trial, the color of the fixation-cross changed into either blue or yellow for 1 s to signal the reward context of the trial. Then, a motion stimulus was presented for 200 ms. The participant had up to 800 ms from stimulus onset to decide the motion direction by pressing the corresponding button. After this 800-ms response window, a number appeared on the screen for 1.5 s to inform the participant how many reward points that they earned from their decisions. The payoff of the two possible motion direction choices was associated with the reward contexts. In one context correct upward motion choices led to more reward points, whereas in the other state correct downward motion choices led to more reward points (Figure 1). The total reward points were converted into US dollar as bonus at the end of the experiment. The participants were unaware of this payoff structure before they began the task. They were simply instructed to decide the motion direction on each trial in order to harvest the most reward points.

Several procedures were implemented in the experimental design to rule out potential sources of decision bias other than reward itself. First, each context was paired with equal numbers of trials in each motion direction and the same levels of motion discriminability, which controlled the prior-induced bias (Green and Swets, 1966). The motion stimulus was a random sample from one of 7 coherence levels (0, ±4, ±12, ±64%) on each trial. Every coherence level repeated 20 times within each state. The total 280 trials (2 reward contexts × 7 coherence levels × 20 repetition) were equally distributed in the 5 scanning runs for each participant. Moreover, the presentation of reward contexts were independent from trial to trial, which rules out the potential confound that participants used the sequential pattern to guide their choices (Glascher et al., 2010; Daw et al., 2011). Finally, the response buttons and the colors of the fixation cross were counter-balanced across participants in order to remove other potential confounds. The participants' choice and the reaction time were recorded on each trial.

### Behavioral data analysis

We applied the hierarchical logistic models to evaluate run-by-run changes of bias and discrimination, using the lme4 package (http://cran.r-project.org/web/packages/lme4/index.html) in R Version 3.0.0 (http://www.r-project.org/). The full model included five exploratory regressors: coherence-level, reward context, run-number, context-by-run, and coherence-by-run interaction. The intercept was taken as a random effect across participants. When testing the learning effects across the five scanning runs, we used a Chi-square test to compare the goodness-of-fit of this full model against the model in which either of the interaction terms was reduced.

Furthermore, we applied computational models to capture the cross-correlation between the feedback on the previous trial and the choice on the next trial. We implemented two reinforcement-learning models with different hypotheses regarding how participants might use their experiences about contexts, choices, and rewards to develop biases in perceptual decision-making. In each case, a logit function was used to generate the probability of binary choices on every trial based on the reward each participant had received so far and the motion stimulus that was presented on the particular trial. The models are described in detail in the next section. Auto-correlation functions were computed from the residuals of the best-fit learning model at the group level using the *acf* function in R (Box et al., 1994; Pinheiro and Bates, 2009) to identify additional factors potentially missing in the model.

### Reinforcement learning models

We assumed that on the *t^th^* trial, an individual chooses probabilistically according to the value difference of each motion direction (*Q_t_*(*m_i_*); a binary variable i, respectively indicating the upward and downward motion) and the perceived motion strength. This relationship can be described by a logit function with the linear combination of the value difference between each motion direction and the perceived motion strength (Green and Swets, 1966; Macmillan and Creelman, 2004). Since the perceived motion strength was monotonic with the physical stimulus (Britten et al., 1993), we used the coherence level on trial t (*S_t_*) to model the contribution of physical stimuli to the choice. The probability of choosing upward motion hence is:

(1) $$P_{t}\;\left(m_{1}\right)\;=\;1/\{1\;+\text{ exp }[\beta_{0}\;*\;\left(Q_{t}\;\left(m_{0}\right)\;-\;Q_{t}\;\left(m_{1}\right)\right)\;+\;\beta_{1}\;*\;S_{t}]\}$$

where the *b*_0_ and *b*_1_ reflected how the choice probability was influenced by the subjective value difference and motion stimulus on the current trial, respectively.

The second part of the model was constructed to simulate how the trial-wise reward feedback was used to estimate the subjective value of each motion direction. Two Q-learning models (Watkins and Dayan, 1992) were separately specified such that the reward prediction error (RPE) was used to update action value differently. The RPE was used to updated the action value pertained to the context in one model and the action value regardless of context in the other model.

On every trial, the context-dependent action value was adjusted by keeping track of the context in which a choice was made. The context-dependent RPE (δ′_*t*_) was computed as the difference between expected and the actual outcomes (*r_t_*) followed by a choice made in the specific context. This error was scaled by a constant learning rate (α′) and was added to the value of each motion direction choice made in this previous context:

(2) $$\begin{array}{l} \;\delta_{t}=\;r_{t}\;-\;Q_{t}\;\left(m_{t}|c_{t}\right),\\ Q_{t+1}\;\left(m_{t}|c_{t}\right)\;=\;Q_{t}\;\left(m_{t}|c_{t}\right)\;+\;\alpha \prime \;*\;\delta \prime_{t} \end{array}$$

where *c_t_* and *m_t_* take binary values respectively for indicating the context on trial t and the motion direction choice made in this context.

A second model was constructed based on the assumption that context was ignored. In this case, the action value was adjusted by adding RPE (δ_*t*_), weighted by a constant learning rate (α), to the value of each motion direction (*Q_t_*(*m_t_*)), regardless of the context where the choice was made:

(3) $$\begin{array}{l} \;\delta_{t}=\;r_{t}\;-\;Q_{t}\;\left(m_{t}\right)\\ Q_{t+1}\;\left(m_{t}\right)\;=\;Q_{t}\;\left(m_{t}\right)\;+\;\alpha \;*\;\delta_{t} \end{array}$$

We used the method of maximum likelihood to obtain parameter estimates for each model. The likelihood that a sequence of choice and feedback (D) was generated by a set of free parameters (θ_*M*_ ∈ {β_0_, β_1_, α}) was the product of Equation (1) over the total trials (Daw, 2011):

(4) $$P\left(\theta_{M}|D\right)\propto P\left(D|\theta_{M}\right)\;=\;\prod_{t}P_{t}\left(m_{1}|\theta_{M}\right)$$

We fit each model's free parameters by minimizing the negative log of Equation (4) with non-linear optimization function (fmin) in the Scipy toolbox for python (http://docs.scipy.org/doc/scipy-0.7.x/reference/optimize.html). We initialized the value of each motion direction choice with the participants' initial bias toward either motion direction, if they showed any in the practice session. Once the best-fit parameters were determined for each participant, the indecision point on each trial was computed by solving the coherence level that yielded equal chance of choosing either motion direction given the rest of the parameters in Equation (1). For model comparison, the Akaike Information Criterion (AIC) was computed by summing the negative log of Equation (4) over all participants (*n* = 23) with the total number of parameters (k) as a penalty term (Akaike, 1974):

(5) $$\text{AIC}=\sum_{n=1}^{23}\sum_{t}{\left(-\log\left(p_{t}(m_{1}|\theta_{M})\right)-k\right)}_{n}$$

### MRI data acquisition

Imaging data were collected using a Siemens Skyra 3T MR scanner. Functional data were collected using a T2^*^-weighted multi-band echo-planar imaging sequence (Moeller et al., 2010) with 60° flip-angle (TR: 2 s; TE: 30 ms; FOV: 256 mm, multi-band acceleration factor: 2, parallel acceleration factor: 2, matrix size: 128). Forty-eight oblique axial slices were collected in interleaved fashion with 2 mm isotropic resolution. To reduce dropout in orbito-frontal cortex, the slices were tilted at a 10–15° angle off of the anterior-commissure-posterior-commissure line and higher-order shimming was applied. T1-weighted anatomical images were collected using an MP-RAGE sequence with 9° flip angle (TR: 1.9 s; TE: 2.43 ms; FOV: 256 mm; Matrix size: 256 × 256, 192 slices; slice thickness: 1 mm).

### Image preprocessing and registration

FMRI data preprocessing was carried out using FSL Version 5.0.1 (FMRIB's Software Library: www.fmrib.ox.ac.uk/). All image time series were aligned with the MCFLIRT tool, and the resulting motion parameters were used to compute frame-wise displacement (FD) and temporal derivative of the root mean square variance over voxels (DVARS) to identify bad time points (*FD* > 0.5; DVARS > 0.5) (Power et al., 2012). The skull was removed from the functional images with the brain extraction tool (BET) and from the structural images using FreeSurfer (https://surfer.nmr.mgh.harvard.edu/). Spatial smoothing was applied using a Gaussian kernel of FWHM 5 mm. The grand-mean intensity was normalized over the entire 4D dataset by a single multiplicative factor, and a high-pass temporal filtering (Gaussian-weighted least-squares straight line fitting, with sigma = 50.0 s). This same high-pass filter was applied to the design matrix for analyzing the fMRI time-series. All functional images were registered to the high resolution structural image using Boundary-Based Registration (BBR) then the high resolution structural image to the MNI-152 2 mm template using the FLIRT linear registration (12 DOF) tool of FSL.

### fMRI analysis

We used multi-stage general linear model (GLM) approach to analyze the brain imaging data, using FSL FEAT (FMRI Expert Analysis Tool) Version 6.00. The first-level model was estimated separately for each run and each participant. All five runs were combined within participant using a fixed-effects model. At the group level, the FLAME 1 mixed-effects model of FSL was applied to all participants. All the statistical maps were corrected by cluster-based random field theory using clusters determined by *Z* > 2.3 and a family-wise error corrected cluster significance threshold of *P* = 0.05 (Worsley, 2001). The statistics maps of all analyses were projected onto the group-averaged brain from this study for visualization.

#### GLM model

The first level of GLM contained parametric modulated regressors to identify the brain mechanisms underlying the acquisition of bias in perceptual decisions as well as nuisance regressors to control for potential confounds. The parametric modulated regressors included (1) the absolute value of the coherence level (duration between the onset and offset of the stimulus), (2) the trial-wise amount of bias (duration between trial onset and stimulus offset), and (3) the reward prediction error derived from both learning models (duration between the onset and offset of the reward feedback). The amount of the bias was derived as the absolute value of the difference between the two choices from the best-fit reinforcement-learning model at the group-level for each participant. All the values of parametric modulated regressors were mean-centered before entering the GLM. Nuisance regressors in the model were (1) a boxcar regressor encoding trial-evoked activity (duration between the onset of the context and the next ITI), (2) a boxcar regressor between the stimulus onset and the time when a key press was detected to control the reaction time (RT), and (3) a confound file including all the motion correction parameters (estimated translation and rotation and their first derivatives, FD, and DVARS) together with single-time-point regressors for each time point that exceeded the FD/DVARS thresholds (which effectively performs “scrubbing” of those time points) (Power et al., 2012). All the regressors except the motion-correction regressors in the first-level model were convolved with a double-gamma hemodynamic response function. Their temporal derivatives were also included in the model to accommodate for potential slice timing differences. Except for the RT regressor that was orthogonalized relative to the regressor for the trial-evoked activity, all other regressors entered the GLM without orthogonalization.

#### Psychophysiological interaction (PPI) analysis

To examine the effect of the value system on the acquisition process of the bias in perceptual decisions, we define a seed region (10-mm sphere around the vmPFC; MNI coordinates: *X* = −6, *Y* = 39, *Z* = −8) according to previous results on value-based decisions (Tom et al., 2007). Likewise, to examine the effect of the fronto-parietal system on the acquisition of bias, we defined seed regions for frontal cortex (MNI coordinates: *X* = −45, *Y* = 21, *Z* = 0) and parietal cortex (MNI coordinates: *X* = −36, *Y* = −39, *Z* = 45) that have been replicated by previous study on the bias in perceptual decisions (Fleming et al., 2010; Summerfield and Koechlin, 2010). The BOLD activation of the seed regions was extracted from each participant's individual brain in each run. For each individual and each run, the neural signal of the seed region was estimated by deconvolving the BOLD signals using the deconvolution algorithm of SPM (Gitelman et al., 2003). The interaction between the seed region and the regressor modulated by trial-wise amount of bias was generated in the neural domain and then reconvolved with hemodynamic function. The first-level design matrix of the PPI analysis was the above-mentioned GLM design matrix with two additional regressors: (1) the raw time course extracted from the seed, and (2) a PPI regressor (the interaction between the amount of bias and the mean BOLD response in the seed region) with duration between the trial onset and the stimulus offset.

## Results

### Behavioral results

**Figure 2:**
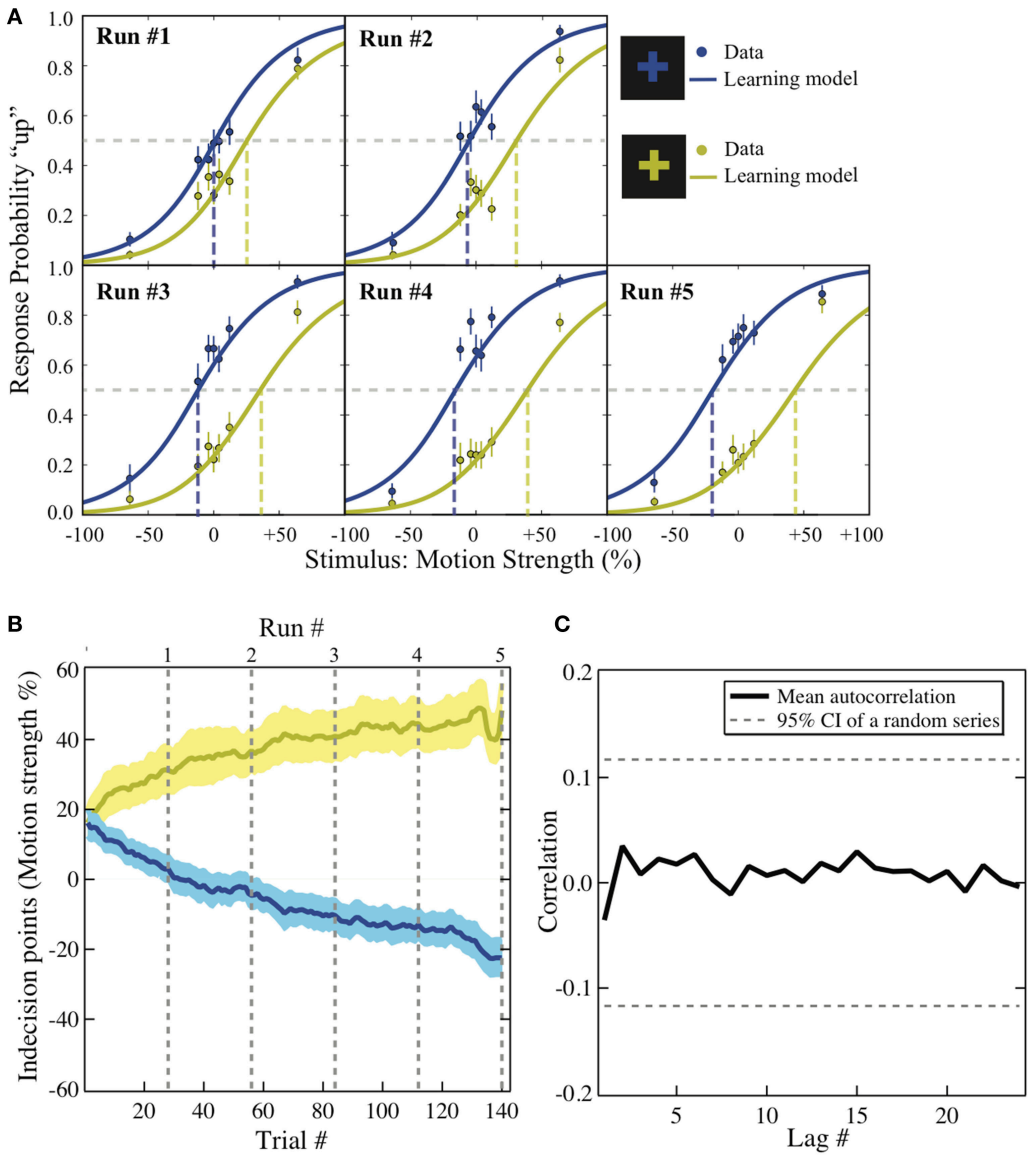
**Behavioral results**. **(A)** *Psychometric function*. The motion strength was plotted against the probability of choosing “up” in each context across the five runs (dots). This change is modeled with logit function in which its intercept reflects the value difference between the two motion directions that has been learned up to the end of each run (solid lines). The dashed lines are the indecision points. Motion strength: the percentage of coherent moving-dots; ±: upward/downward motion. Error bars: ±1 s.e.m. **(B)** *The bias acquisition process*. The trial numbers are plotted against the indecision points estimated by the reinforcement-learning model using individuals' data. Solid lines: group mean. Shaded areas: ±1 s.e.m. Dashed lines: the end of each run. Colors: corresponding to the reward context as illustrated in **(A)**. **(C)** *The autocorrelation functions*. The correlation estimates using the residuals from the context-based learning model is plotted against each lag. Solid lines: group average. Dashed lines: 95% confidence interval of the autocorrelation estimated from a random series with the same number of trials (a total of 280 trials).

To visualize how the decisions changed as the experiment unfolded, the group average of the choice probability in each context is plotted against the coherence level across the five runs (Figure 2A; solid dots). We first applied hierarchical logistic regression models to identify the factors driving the changes over the experiment. The intercept of each of these models was taken as a random effect across individual participants. The full model assumes that both the participants' ability to discriminate motion direction *and* their preference for one of the motion directions changes from run to run in the experiment. If either of these factors is not constant over the experiment, removing either term from the full model should significantly reduce the model fit to the data. We used a Chi-square test for model comparison to evaluate whether the drop in goodness-of-fit between the full and the reduced models reaches significance. We find that the interaction between coherence level and run number can be eliminated from the full model (χ^2^ (4) = 6.15, *p* = 0.1881 > α = 0.05), suggesting that the participants' ability to discriminate motion direction did not change significantly over the entire experiment. However, removing the interaction between the reward context and the run number from the full model significantly reduces the model fit to the data (χ^2^ (4) = 72.22, *p* < 0.0001), suggesting that the degree of bias did change across runs of the experiment. In other words, the best-fit model contains only the interaction between the run number and the reward context.

We further examined how the trial-wise reward feedback can account for the changes of the intercepts in perceptual decisions from trial to trial. The first hypothesis is that in addition to the motion stimulus presented on the current trial, the participants may also utilize the previous reward that had been received in a particular context to guide the next choice that would be made in the same context (formalized in Equation 2). Alternatively, the participants may simply adjust their perceptual choice according to the reward obtained directly on the previous trial (independent of context) as well as the strength of the motion stimulus that is presented in front of them (formalized in Equation 3). These two possibilities were evaluated through model comparison. As listed in Table 1, we find that the reinforcement-learning model that associates previous reward and the next choice in the same context (Equation 2) fits the data better than the learning model that ignores the context (Equation 3). Furthermore, combining each participant's data and the best parameters in this reinforcement-learning model (Equations 1 and 2), we are able to infer the trajectory of the indecision points from trial to trial (Figure 2B) and reproduce the choice probability up to the final trial of each run (solid lines in Figure 2A) at the group level.

**Table 1:** **Best-fitting parameter estimates**.

**Parameters**	**Learning model I (contextual action-outcome association; Equations 1 and 2)**	**Learning model II (action-outcome association; Equations 1 and 3)**	**Hierarchical logistic regression (context by run number interaction)**
*b*_0_	0.81 (0.39, 1.23)	1.03 (0.01, 1.44)	–
α	0.02 (0.009, 0.03)	0.01 (0.006, 0.02)	–
*b*_1_	4.01 (3.54, 4.80)	3.54 (2.47, 4.11)	–
AIC	**6672.9**	7770.24	6962
N(fit)/N(total)	21/23	2/23	–

*The parameter values are shown as median and the interquartile range (25th, 75th percentile) across participants. Also shown are the proportions of participants whose data are better fit by each of the learning model. Bold fonts, the best-fit model; N, the number of participants; AIC, Akaike's Information Criterion*.

One may suspect that the participant simply applied pre-existing knowledge about perceptual uncertainty and reward to make a choice rather than adjusting their choice from trial to trial (Whiteley and Sahani, 2008). If this is the case, the above-mentioned hierarchical logistic regression model that treats each choice as an independent observation should fit the data better than the reinforcement-learning model that accounts for the cross-correlation between previous decision outcome and the next choice made in the same context. The result of model comparison indicates that the reinforcement-learning model does provide a better fit to the data than the hierarchical logistic regression model that can possibly best account for the obtained data (Table 1). This suggests that the bias in perceptual decisions was acquired through trial-wise adjustment according to contextual feedback in our task.

One may also suspect that in addition to the context and reward association, the previous choice (Lau and Glimcher, 2005) or even the sequential structure of stimulus types (Cho et al., 2002) alone could contribute to the observed bias. We calculated the average correlation between residuals that are lagged behind a certain number of trials (autocorrelation functions) after the context and reward association has been accounted for in the reinforcement-learning model. If the response-by-response or stimulus-by-stimulus structure was an additional source of bias in our experiment, we should observe that some of the autocorrelations in the residuals are significantly non-zero since these factors were ignored in the learning model. As illustrated in Figure 2C, we compared the autocorrelation functions estimated from the residuals against those estimated from an independent random process with the same number of trials. The dashed horizontal lines show the 95% confidence intervals for autocorrelations expected from an independent random process. All the autocorrelations estimated from the residuals fall well within this confidence interval. This suggests that there is no appreciable temporal structure in the residuals after the context and reward association was taken into account.

### Neuroimaging results

#### The neural correlates of the acquired bias in perceptual decisions

**Figure 3:**
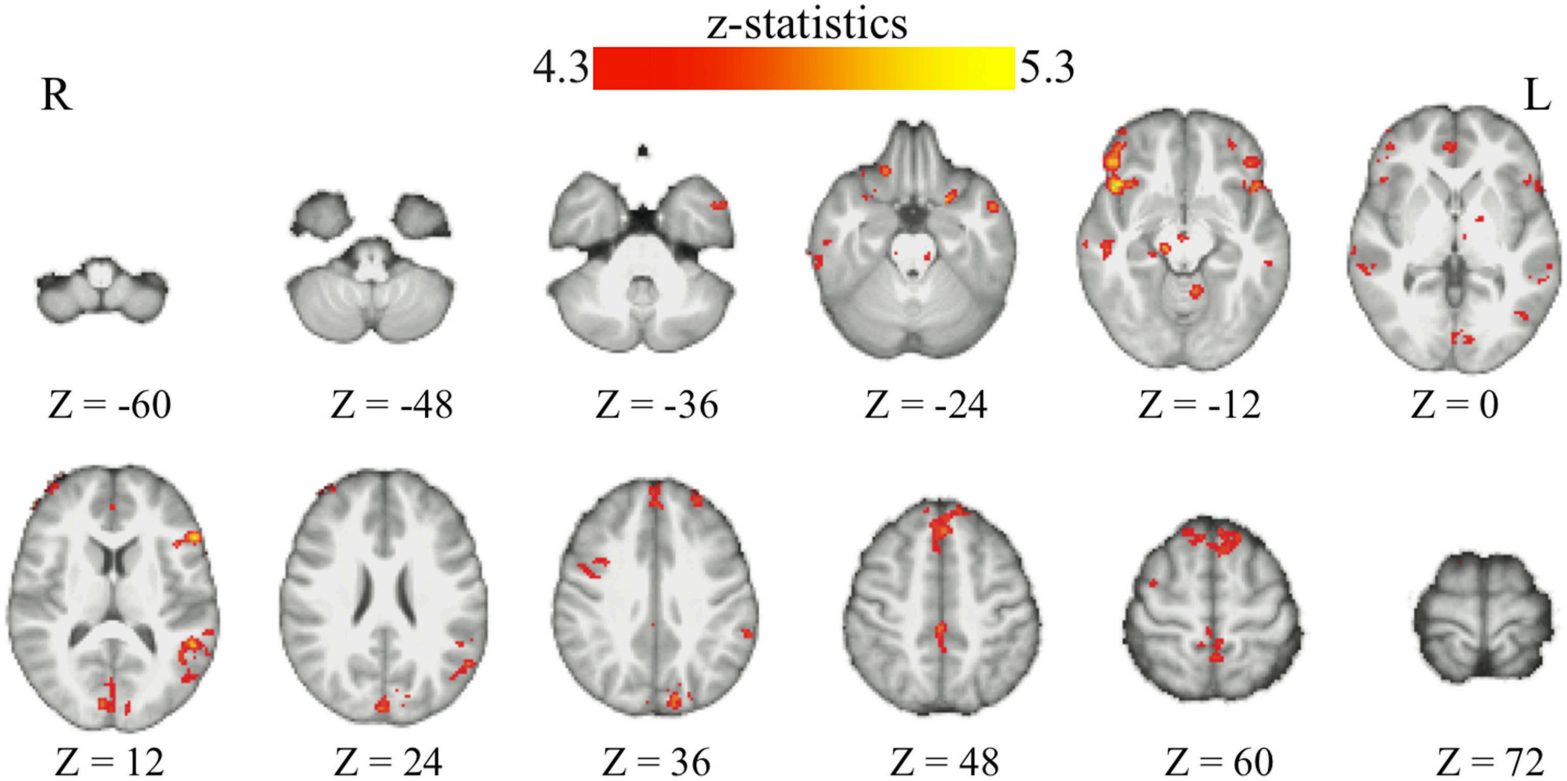
**The acquired bias in the bran**. The maps show the brain areas whose activation positively correlates with the amount of acquired bias on each trial. No brain areas negatively correlate with this signal after the whole-brain correction of multiple comparisons. All maps are presented at *p* < 0.05 whole-brain corrected using cluster-based Gaussian random field and overlaying on the mean anatomical images from the group of participants. R, right hemisphere; L, left hemisphere; Z, the MNI coordinate of the axial slice.

Through the analyses of the behavioral data, we found that the amount of the bias in perceptual decisions on each trial can be modeled as the subjective value difference between the two motion directions at that particular time point of the reinforcement-learning process. If the bias in perceptual decisions is acquired through the dopaminergic system as in value-based decision-making (Nomoto et al., 2010; Rao, 2010; Bogacz and Larsan, 2011), we would expect to see the activation of the value-based decision network (Bartra et al., 2013) to positively correlate with the amount of bias that has been acquired prior to each trial of the experiment. Using the absolute value difference between the two motion directions in each context (estimated from the best-fit reinforcement-learning model) as a parametric modulated regressor, we find that the activation of vmPFC and midbrain dopaminergic areas positively correlates with the amount of bias acquired in each reward context (Figure 3; Table 2). Moreover, we find that the amount of bias acquired through reinforcement has a wider influence on the perceptual decision networks in the brain (Figure 3; Table 2). In addition to the fronto-parietal cortices observed using explicit instructions about reward (Fleming et al., 2010; Summerfield and Koechlin, 2010; Mulder et al., 2012), we also observe that the activation in the visual and motor cortices positively correlate with the amount of acquired bias from trial to trial. No regions showed negative correlations with this regressor after whole-brain correction.

**Table 2:** **GLM results**.

**Effect**	**Brain regions**	**Cluster size (voxels)**	***p*-value**	***z*-value**	**MNI coordinates**		
					*X*	*Y*	*Z*
Acquired bias	Frontal orbital cortex	1336	<0.0001	4.13	46	20	−12
	Superior frontal gyrus	949	<0.0001	3.37	0	34	50
	Inferior frontal gyrus	766	<0.0001	3.77	−54	24	12
	Posterior supramarginal gyrus	729	<0.0001	3.98	−52	−46	10
	Superior lateral occipital cortex	692	<0.0001	3.48	−12	−84	38
	Posterior middle temporal gyrus	364	<0.0001	3.25	62	−30	−16
	Postcentral gyrus	262	<0.0001	3.55	2	−34	52
	Brain-stem	237	<0.0001	3.89	14	−22	−14
	Middle frontal gyrus	167	<0.0001	3.13	38	0	64
	Precentral gyrus	163	<0.0001	3.17	44	0	34
	Frontal medial cortex	105	0.005	2.95	0	46	2
	Posterior superior temporal gyrus	100	0.007	3.16	58	−34	4
	Frontal pole	94	0.011	3.40	−28	52	34
	Temporal pole	91	0.014	3.26	−54	4	−24
Contextual RPE	Superior lateral occipital cortex	3635	<0.0001	4.28	50	−62	24
	Posterior supramarginal gyrus	1596	<0.0001	4.39	−50	−48	52
	Superior frontal gyrus	1395	<0.0001	4.26	−16	18	52
	Posterior middle temporal gyrus	1189	<0.0001	4.26	−62	−40	−8
	Middle frontal gyrus	1134	<0.0001	3.69	38	26	52
	Frontal medial cortex	1065	<0.0001	4.43	2	44	−4
	Posterior cingulate gyrus	1063	<0.0001	3.80	0	−36	42
	Frontal pole	443	<0.0001	3.58	50	44	20
	Inferior lateral occipital cortex	308	<0.0001	3.15	42	−78	−2
	Right caudate	270	<0.0001	4.42	12	12	4
	Cerebellum	234	<0.0001	4.15	−40	−68	−38
	Frontal pole	194	<0.0001	3.85	−20	36	−16
	Left caudate/Accumbens	170	<0.0001	4.01	−10	10	0
	Frontal orbital cortex	100	0.002	3.39	−26	22	−20
	Anterior parahippocampal gyrus	67	0.037	3.37	20	−2	−26
Context-free RPE	Occipital pole	179	<0.0001	3.49	28	−96	16
	Pre-central gyrus	101	0.002	3.28	−48	−18	52

#### Learning signals for the acquisition of bias in perceptual decisions

Human fMRI studies have identified that when individuals use reward feedback to adjust the subjective value of each possible action, the signal pertaining to the difference between the expected and actual reward (reward prediction error, RPE) is represented in ventral striatum, a major target of midbrain dopaminergic neurons (Pagnoni et al., 2002). Recent findings further distinguish that when multiple reward contexts are involved in a task, the activation of fronto-parietal cortices in addition to the ventral striatum correlates with the PE signals (Glascher et al., 2010; Daw et al., 2011). Following the same framework, our reinforcement-learning model assumes that the bias in perceptual decisions is acquired from adjusting the subjective value of each action (motion directions) in the context using prediction errors. Based on the above-mentioned findings, we suspect that our task may involve two types of RPE signals. One is derived from the action-outcome association; the other is derived from the action-outcome association that is contingent on a specific context. We used two learning models (see Materials and Methods section for details) to generate each type of RPE as parametric modulated regressors in order to search for its neural correlates.

**Figure 4:**
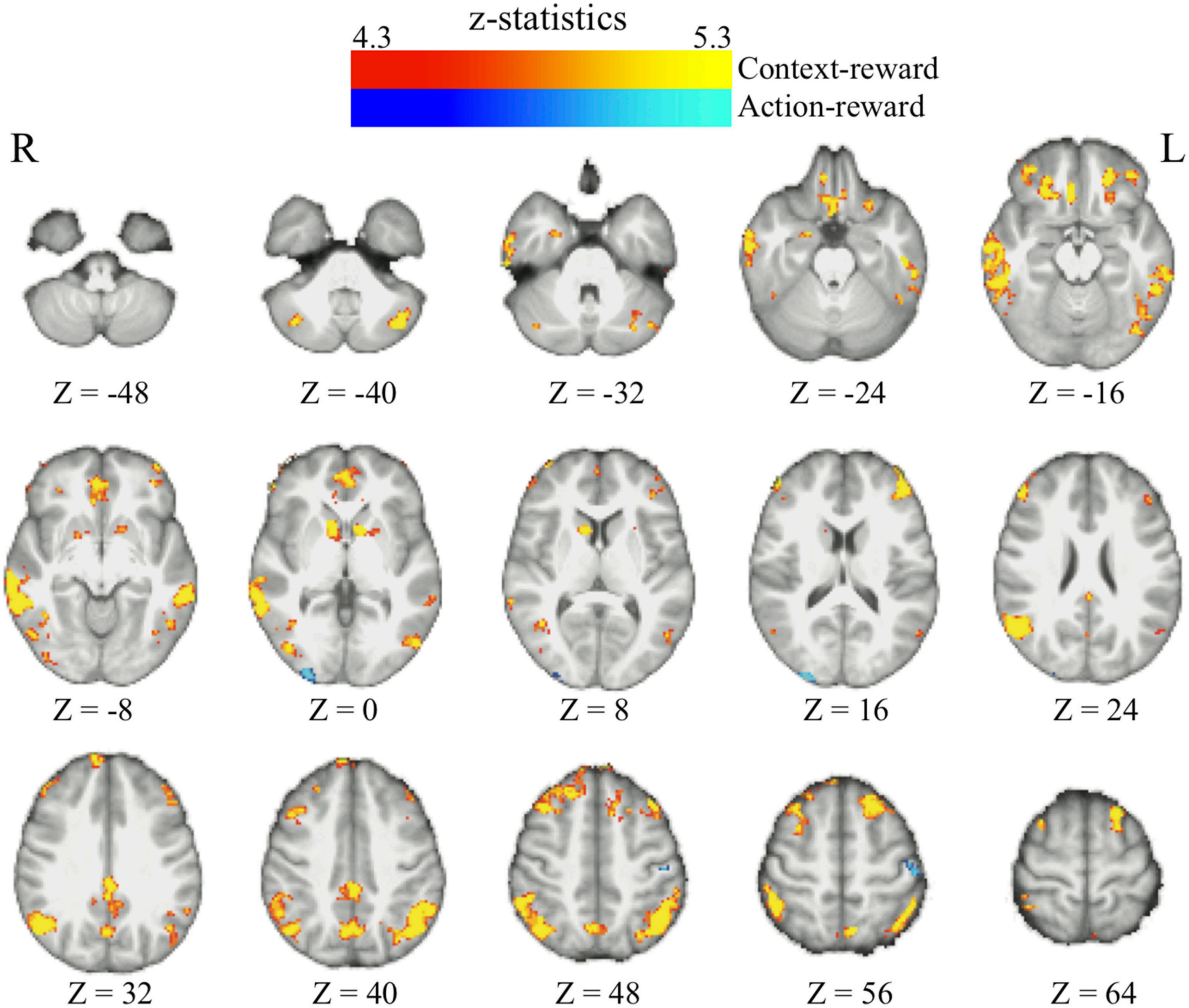
**The reward prediction errors**. The brain maps show the regions whose activation positively correlates with the two types of reward prediction error signals in perceptual decisions after adjusting one type over the other. All maps are presented at *p* < 0.05 whole-brain corrected using cluster-based Gaussian random field and overlaying on the mean anatomical images from the group of participants. Red-Yellow, context-based RPE; Blue-Light-blue, context-free RPE; R, right hemisphere; L, left hemisphere; Z, the MNI coordinate of the axial slice.

We found that in perceptual decisions, both the context-based and context-free RPE regressor reveals a similar pattern of brain activation. Activation in ventral striatum, fronto-parietal, visual, and motor cortices positively correlated with both RPE signals when each of the regressors was modeled separately in a GLM. This pattern is consistent with the meta-analysis result of RPE signals in the brain elicited by economic decisions (Garrison et al., 2013). However, distinct patterns were revealed when both of the regressors were presented together in the same GLM *without orthognization*. The RPE derived from the contextual action-outcome association positively correlated with the ventral striatum, the fronto-parietal cortices, and the posterior and anterior cingulate gyrus (Figure 4, yellow-red; Table 2). In contrast, PE signals from the context-free RL model positively correlated with activity in the visual and motor cortices (Figure 4, blue-light blue; Table 2). Generally, collinearity between regressors weakens the statistical power to detect the correlation between dependent variables and the independent variable. Given this situation, the significant correlation reported here implies that these effects may be quite large. Although interesting, testing whether the different patterns suggest dissociated mechanisms is beyond the limitation of our statistical methods.

One may argue that the activation pattern that uniquely correlates with the context-based RPE simply reflects the level of surprise (Ding and Gold, 2010; Gottlieb, 2012) that has nothing to do with whether the actual outcomes are better or worse than one's expectation. We therefore added the absolute value of this RPE (i.e., unsigned RPE) into the above-mentioned model to account for the level of surprise. The signed context-based RPE still elicits the same activation pattern (whole-brain corrected) as described above after the level of surprise is controlled in the statistical model. No brain regions show activation that correlates with the level of surprise (i.e., unsigned RPE) after whole-brain correction for multiple comparisons, hence ruling out this potential confound in our results.

#### The changes in functional connectivity as the bias grows

We further tested the hypothesis that bias occurs in perceptual decision-making when the information about reward modulates functional connectivity within perceptual decision networks (Liston and Stone, 2008; Bogacz and Larsan, 2011). One theory suggests that the frontal and parietal cortices may provide top-down influence to selectively facilitate the visual representation in favors for the higher-valued option (Summerfield et al., 2006; Fleming et al., 2010). This assumption implies that we should expect to see increased functional connectivity between the frontal/parietal and the visual cortices as individuals acquire bias in perceptual decisions. Furthermore, our learning model assumes that the bias in perceptual decisions at the behavioral level reflects the subjective value difference between each action (i.e., choosing one of the two motion directions) in the current context. This assumption then suggests that the functional connectivity between the value and motor system should increase over time, as the participants learn more about the value of each option.

We use psychophysiological interaction (PPI) analysis to examine how the interaction among these functionally specialized networks changes as individuals acquire bias in perceptual decisions. The above-mentioned brain areas (i.e., frontal, parietal, and value systems) are selected according to the previous literature and used as the seed regions in three independent PPI analyses. We centered the seed regions of frontal and parietal cortices at the MNI coordinates in which the BOLD signals have been repeatedly shown to correlate with the magnitudes of reward-induced bias in perceptual decisions (Fleming et al., 2010; Summerfield and Koechlin, 2010). The vmPFC were selected with the MNI coordinates where its peak response to reward was observed in value-based decisions (Tom et al., 2007).

**Figure 5:**
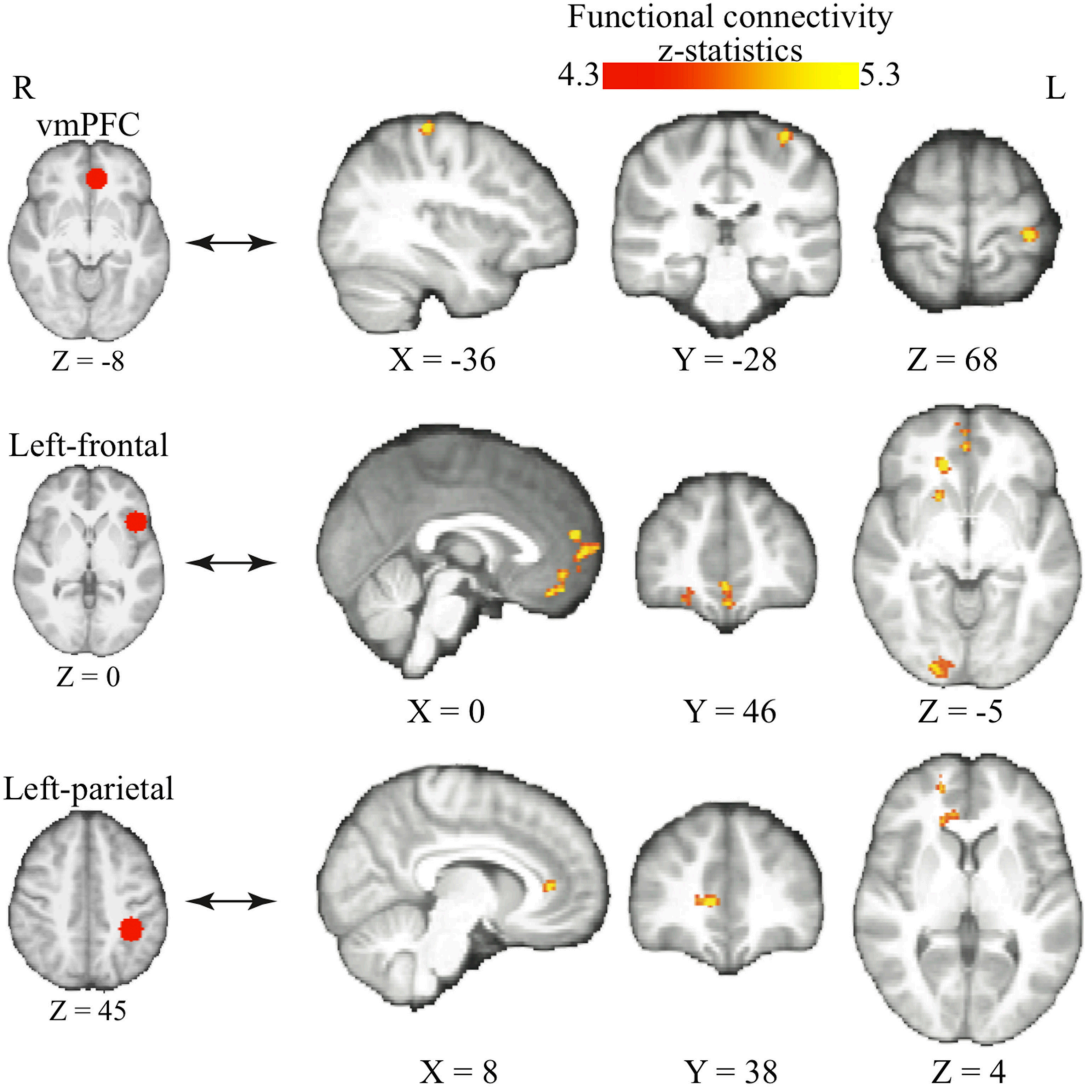
**Three functional connectivity patterns underlying the growth of bias**. The left panel shows the seed regions that were used in the psychophysiological interaction analyses. These seed regions were selected according to the literature and centered at the MNI coordinates (vmPFC: [−6, 39, −8]; left-frontal: [−45, 21, 0]; left-parietal: [−36, −39, 45]) with the radius of 10 mm. The brain maps on the right panel show the areas that positive correlate with the interaction between each of the seed regions and the amount of acquired bias on each trial. The statistical maps are corrected for multiple comparisons at the whole-brain level using cluster-based Gaussian random field correction at *P* < 0.05 and overlaying on the mean anatomical images from the group of participants. R, right hemisphere; L, left hemisphere; X, Y, Z, the MNI coordinate of the brain slice.

We find that as people acquire bias in perceptual decision, the information about reward formed three different sets of functional connectivity patterns that were more complicated than expected based upon the current literature. The information about reward actually was integrated into three distinct functional connectivity patterns among visual, motor, and cognitive control systems (Figure 5; Table 3). First, using the left frontal cortex as a single seed region, its connectivity with vmPFC, ventral striatum, and visual cortex increased as bias grew. Second, using the left parietal cortex as the seed region, its connectivity with ACC increased as bias grew. Finally, using vmPFC as the seed region of the value system in the PPI analysis, we find that the task-related interaction between vmPFC and left motor cortex increases as the amount of bias increases over the experiment (notice that our participants used their right hand to press response buttons in the experiment).

**Table 3:** **PPI results**.

**Seed regions**	**Co-activating brain regions**	**Cluster size (voxels)**	***p*-value**	***z*-value**	**MNI coordinates**		
					***X***	***Y***	***Z***
vmPFC (MNI coordinates [x, y, z] = [−6, 39, 8])	Pre-central gyrus	56	0.037	3.44	−36	−28	68
(Left) Frontal cortex (MNI coordinates [x, y, z] = [−45, 21, 0])	Occipital pole	325	<0.0001	3.27	22	−92	−2
	Paracingulate gyrus	131	<0.0001	3.42	0	54	16
	Frontal medial cortex	117	<0.0001	3.24	−4	38	−20
	Occipital fusiform gyrus	90	0.003	3.10	−48	−70	−26
	Right putamen/Caudate	63	0.039	3.09	18	16	−4
(Left) Parietal cortex (MNI coordinates [x, y, z] =[−36, −39, 45])	Anterior cingulate cyrus	98	0.001	3.07	10	38	6
	Paracingulate gyrus	71	0.009	3.37	18	52	2

## Discussion

In this report, we demonstrated a neurocomputational basis for the development of bias in perceptual decision-making. We found that in perceptual decisions, individuals constantly learn the values of the two potential perceptual choices (e.g., the two motion directions in our task) by associating the deviation between the expected and actual outcome (RPE) with each reward context. When the context is clearly indicated, a perceptual choice reflects the sum of the present sensory information and the expected value difference between the two options that has been learned so far. At the neural level, the activation of ventral striatum, frontal, and parietal cortices is positively correlated with the contextual RPE derived from modeling each individual's performance over the experiment. Moreover, through this reinforcement learning mechanism implemented in the dopaminergic system, over the experiment, the value signals became increasingly integrated over the learning period with neural systems involved in action, stimulus evaluation, and cognitive control that gradually influence ones' perceptual decisions. These results reveal the shared neural mechanism between perceptual and economic decisions, and highlight the involvement of multiple control networks that are sensitive to reward during perceptual decision-making.

There is increasing convergence across different neuroscience methods and across species regarding the involvement of reinforcement learning processes in perceptual decisions. Most of these findings focus on the improvement in individuals' ability to detect or discriminate sensory information, or perceptual learning (Law and Gold, 2008; Kahnt et al., 2011). Here, we extend this line of research by showing that bias in perceptual decision-making can also be acquired through similar learning mechanism. Given the commonality between our findings and the findings in perceptual learning, one may suspect that the RPE signals that we report in ventral striatum, frontal, and parietal cortices may simply reflect perceptual learning (Kahnt et al., 2011) rather than the development of bias. However, we found that participants' ability to discriminate the motion direction did not improve significantly over the experiment. In addition, our experiment may be too short to elicit the effect of perceptual learning, which usually requires more than a thousand trials (Law and Gold, 2008; Kahnt et al., 2011). This mismatched temporal dynamic between perceptual learning and the acquisition of bias further rules out this alternative interpretation.

Our findings also speak to the great interest in the literature regarding the integration of reward and sensory information in the brain during perceptual decisions. Perceptual decision-making involved neural processes of accumulating evidence (Shadlen and Newsome, 2001), selecting action (Filimon et al., 2013), and central execution such as speed-accuracy trade-offs or decision caution (Palmer et al., 2005; Forstmann et al., 2010). Theories and evidence suggests that information about reward influences the higher level of perceptual decision hierarchy (frontal and parietal cortices) and the downstream visual and motor processes (Liston and Stone, 2008; Serences, 2008; Fleming et al., 2010; Summerfield and Koechlin, 2010; Mulder et al., 2012). Our finding extends this view by showing how functional connectivity patterns changed as individuals learned to use the reward information to guide perceptual choices from trial to trial. According to our PPI results, the influences of reward on these perceptual decision components speak against a single top-down facilitation process within the perceptual decision hierarchy (Fleming et al., 2010; Summerfield and Koechlin, 2010). The information of reward integrates into sensory evaluation through the functional connection among visual, vmPFC, and lateral prefrontal cortices. Its influence on motor planning is evident in the increased functional connectivity between vmPFC and motor cortex rather than direct effects from lateral PFC.

Since perceptual decisions are usually analyzed under the framework of drift-diffusion process, one obvious question is how our finding relates to this tradition. In fact, many theoretical approaches have been proposed to account for the function of basal ganglia and its contribution to the learning of the bias in perceptual decisions in terms of drift-diffusion process (Rao, 2010; Bogacz and Larsan, 2011). However, it is very challenging to empirically test these theories at the whole brain level. The difficulty is that bias induced by reward usually is weak in human studies using reaction time tasks (Mulder et al., 2012) because of the speed-accuracy trade-off (Maddox and Bohil, 1998; Simen et al., 2009). In order to investigate the acquisition process of the bias in perceptual decisions, we applied a decision deadline to boost the effect of reward-induced bias since this type of bias usually occurs in fast choices and suggests the influence on the starting point of the drift-diffusion process (Summerfield and Koechlin, 2010; Mulder et al., 2012; White and Poldrack, 2014). Using this manipulation, we present the first empirical evidence showing the role of basal ganglia in the acquisition of the bias in perceptual decisions and three functional connectivity patterns pertaining to express this acquired bias in the human brain. However, the drawback of this speeded-response manipulation is that it limits the interpretation of our finding to the speeded decisions and prevents us from applying the drift-diffusion model to the present data. Future work should focus on identifying the neural mechanisms by which bias presents as individuals freely adjust reaction time in order to maximize reward.

Finally, one may be interested in whether the participants were aware of the payoff structure or not during the experiment. We prompted the participants to report their strategies after finishing the experiment. However, most of them simply said that they did their best in classifying the motion direction and some trials were very difficult. Some of them felt that the payoffs somehow depended on the color of the cues; however, no participants verbalized the specific rule. Determining whether the participants used explicit or implicit knowledge about the payoff structure is beyond the scope of this paper, given our lack of sensitive assessments of explicit knowledge.

In conclusion, the present study shows that bias in perceptual decision-making can arise from the neural mechanisms that learn the association between contexts and choice outcomes. The learning signals (contextual RPE) are observed in ventral striatum, frontal, and parietal cortices. The acquired bias mirrors the learned value difference between each perceptual choice at the behavioral level and correlates with multiple connectivity patterns as such bias grows. This indicates that the information about value contributes to bias in perceptual decision-making through modulating multiple systems (perceptual-, decision-, and action-networks) instead of a single top-down process, as usually suggested in the literature. Our results hence demonstrate the pervasive effects of reinforcement learning mechanisms on the whole-brain connectivity by which the subjective expectation of reward colors the process of objective evidence in decision-making.

### Conflict of interest statement

The authors declare that the research was conducted in the absence of any commercial or financial relationships that could be construed as a potential conflict of interest.

## References

[B1] Akaike H. (1974). A new look at the statistical model identification. IEEE Trans. Automat. Contr. 19, 716–723 10.1109/TAC.1974.1100705

[B2] Bartra O. McGuire J. T. Kable J. W. (2013). The valuation system: a coordinate-based meta-analysis of BOLD fMRI experiments examining neural correlates of subjective value. Neuroimage 76, 412–427. 10.1016/j.neuroimage.2013.02.063 23507394 PMC3756836

[B4] Bogacz R. Larsan T. (2011). Integration of reinforcement learning and optimal decision-making theories of the basal ganglia. Neural Comput. 23, 817–851. 10.1162/NECO_a_00103 21222528

[B5] Box G. Jenkins G. M. Reinsel G. (1994). Time Series Analysis: Forecasting and Control, 3rd Edn. Englewood Cliffs, NJ: Wiley.

[B6] Brainard D. H. (1997). The psychophysics toolbox. Spat. Vis. 10, 433–436. 10.1163/156856897X00357 9176952

[B7] Britten K. H. Shadlen M. N. Newsome W. T. Movshon J. A. (1993). Responses of neurons in macaque MT to stochastic motion signals. Vis. Neurosci. 10, 1157–1169. 10.1017/S0952523800010269 8257671

[B8] Cho R. Y. Nystorm L. E. Brown E. T. Jones A. D. Braver T. S. Holmes P.. (2002). Mechanisms underlying dependencies of performance on stimulus history in a two-alternative forced-choice task. Cogn. Aff. Behav. Neurosci. 4, 283–299. 10.3758/CABN.2.4.283 12641174

[B9] Daw N. D. (2011). Trial-by-trial data analysis using computational models, in Decision Making, Affect, and Learning: Attention and Performance XXIII, eds Delgado M. R. Phelps E. A. Robbins T. W. (New York, NY: Oxford University Press), 1–37 10.1093/acprof:oso/9780199600434.003.0001

[B10] Daw N. D. Gershman S. J. Seymour B. Dayan P. Dolan R. J. (2011). Model-based influences on humans' choices and striatal prediction errors. Neuron 69, 1204–1215. 10.1016/j.neuron.2011.02.027 21435563 PMC3077926

[B11] Ding L. Gold J. I. (2010). Caudate encodes multiple computations for perceptual decisions. J. Neurosci. 30, 15747–15759. 10.1523/JNEUROSCI.2894-10.2010 21106814 PMC3005761

[B12] Ding L. Gold J. I. (2013). The Basal ganglia's contributions to perceptual decision making. Neuron 79, 640–649. 10.1016/j.neuron.2013.07.042 23972593 PMC3771079

[B13] Edwards W. (1965). Optimal strategies for seeking information: models for statistics, choice reaction times, and human information processing. J. Math. Psychol. 2, 312–329 10.1016/0022-2496(65)90007-6

[B14] Feng S. Holmes P. Rorie A. Newsome W. T. (2009). Can monkeys choose optimally when faced with noisy stimuli and unequal rewards? PLoS Comput. Biol. 5:e1000284. 10.1371/journal.pcbi.1000284 19214201 PMC2631644

[B15] Filimon F. Philiastides M. G. Nelson J. D. Kloosterman N. A. Heekeren H. R. (2013). How embodied is perceptual decision making? Evidence for separate processing of perceptual and motor decisions. J. Neurosci. 33, 2121–2136. 10.1523/JNEUROSCI.2334-12.2013 23365248 PMC6619122

[B16] Fleming S. M. Whiteley L. Hulme O. J. Sahani M. Dolan R. J. (2010). Effects of category-specific costs on neural systems for perceptual decision-making. J. Neurophysiol. 103, 3238–3247. 10.1152/jn.01084.2009 20357071 PMC2888245

[B17] Forstmann B. U. Anwander A. Schaefer A. Neumann J. Brown S. Wagenmakers E. J. (2010). Cortico-striatal connections predict control over speed and accuracy in perceptual decision making. Proc. Natl. Acad. Sci. U.S.A. 107, 15916–15920 10.1073/pnas.1004932107 20733082 PMC2936628

[B18] Garrison J. Erdeniz B. Done J. (2013). Prediction error in reinforcement learning: a meta-analysis of neuroimaging studies. Neurosci. Biobehav. Rev. 37, 1297–1310. 10.1016/j.neubiorev.2013.03.023 23567522

[B19] Gitelman D. R. Penny W. D. Ashburner J. Friston K. J. (2003). Modeling regional and psychophysiologic interactions in fMRI: the importance of hemodynamic deconvolution. Neuroimage 19, 200–207. 10.1016/S1053-8119(03)00058-2 12781739

[B20] Glascher J. Daw N. Dayan P. O'Doherty J. P. (2010). States versus rewards: dissociable neural prediction error signals underlying model-based and model-free reinforcement learning. Neuron 66, 585–595. 10.1016/j.neuron.2010.04.016 20510862 PMC2895323

[B21] Glimcher P. W. (2011). Understanding dopamine and reinforcement learning: the dopamine reward prediction error hypothesis. Proc. Natl. Acad. Sci. U.S.A. 108, 15647–15654. 10.1073/pnas.1014269108 21389268 PMC3176615

[B22] Gold J. I. Ding L. (2013). How mechanisms of perceptual decision-making affect the psychometric function. Prog. Neurobiol. 103, 98–114. 10.1016/j.pneurobio.2012.05.008 22609483 PMC3445702

[B23] Gottlieb J. (2012). Attention, learning, and the value of information. Neuron 76, 281–295. 10.1016/j.neuron.2012.09.034 23083732 PMC3479649

[B24] Green D. M. Swets J. A. (1966). Signal Detection Theory and Psychophysics. New York, NY: Wiley. Reprinted by Krieger, Huntington, 1974.

[B25] Kahnt T. Grueschow M. Speck O. Haynes J.-D. (2011). Perceptual learning and decision-making in human medial frontal cortex. Neuron 70, 549–559. 10.1016/j.neuron.2011.02.054 21555079

[B26] Lau B. Glimcher P. W. (2005). Dynamic response-by-response models of matching behavior in rhesus monkeys. J. Exp. Anal. Behav. 84, 555–579. 10.1901/jeab.2005.110-04 16596980 PMC1389781

[B27] Law C.-T. Gold J. I. (2008). Neural correlates of perceptual learning in a sensory-motor, but not a sensory, cortical area. Nat. Neurosci. 11, 505–513. 10.1038/nn2070 18327253 PMC2424192

[B28] Lee D. Seo H. Jung M. W. (2012). Neural basis of reinforcement learning and decision making. Ann. Rev. Neurosci. 35, 287–308. 10.1146/annurev-neuro-062111-150512 22462543 PMC3490621

[B29] Liston D. B. Stone L. S. (2008). Effects of prior information and reward on oculomotor and perceptual choices. J. Neurosci. 28, 13866–13875. 10.1523/JNEUROSCI.3120-08.2008 19091976 PMC6671904

[B30] Macmillan N. A. Creelman D. C. (2004). Detection Theory: A User's Guide, 2nd Edn. Mahwah, NJ: Lawrence Erlbaum.

[B31] Maddox W. T. (2002). Toward a unified theory of decision criterion learning in perceptual categorization. J. Exp. Anal. Behav. 78, 567–595. 10.1901/jeab.2002.78-567 12507020 PMC1284916

[B32] Maddox W. T. Bohil C. J. (1998). Base-rate and payoff effects in multidimensional perceptual categorization. J. Exp. Psychol. 24, 1459–1482. 10.1037/0278-7393.24.6.1459 9835061

[B33] Moeller S. Yacoub E. Olman C. A. Auerbach E. Strupp J. Harel N. (2010). Multiband multislice GE-EPI at 7 tesla, with 16-fold acceleration using partial parallel imaging with application to high spatial and temporal whole-brain fMRI. Mag. Res. Med. 63, 1144–1153 10.1002/mrm.22361 PMC2906244 20432285

[B34] Montague P. R. Berns G. S. (2002). Neural economics and the biological substrates of valuation. Neuron 36, 265–284. 10.1016/S0896-6273(02)00974-1 12383781

[B35] Mulder M. J. Wagenmakers E. J. Ratcliff R. Boekel W. Forstmann B. U. (2012). Bias in the brain: a diffusion model analysis of prior probability and potential payoff. J. Neurosci. 32, 2335–2343. 10.1523/JNEUROSCI.4156-11.2012 22396408 PMC6621823

[B36] Newsome W. T. Britten K. H. Movshon J. A. (1989). Neuronal correlates of a perceptual decision. Nature 341, 52–54. 10.1038/341052a0 2770878

[B37] Nomoto K. Schultz W. Watanabe T. Sakagami M. (2010). Temporally extended dopamine responses to perceptually demanding reward-predictive stimuli. J. Neurosci. 30, 10692–10702. 10.1523/JNEUROSCI.4828-09.2010 20702700 PMC3297489

[B38] Pagnoni G. Zink C. F. Montague P. R. Berns G. S. (2002). Activity in human ventral striatum locked to errors of reward prediction. Nat. Neurosci. 5, 97–98. 10.1038/nn802 11802175

[B39] Palmer J. Huk A. C. Shadlen M. N. (2005). The effect of stimulus strength on the speed and accuracy of a perceptual decision. J. Vis. 5, 376–404. 10.1167/5.5.1 16097871

[B40] Pelli D. G. (1997). The VideoToolbox software for visual psychophysics: transforming numbers into movies. Spat. Vis. 10, 437–442. 10.1163/156856897X00366 9176953

[B41] Pinheiro J. E. Bates D. (2009). Mixed-Effects Models in S and S-PLUS (Statistics and Computing), 1st Edn. New York, NY: Springer.

[B42] Power J. D. Barnes K. A. Snyder A. Z. Schlaggar B. L. Petersen S. E. (2012). Spurious but systematic correlations in functional connectivity MRI networks arise from subject motion. Neuroimage 59, 2142–2154 10.1016/j.neuroimage.2011.10.018 22019881 PMC3254728

[B43] Rao R. P. N. (2010). Decision making under uncertainty: a neural model based on partially observable markov decision processes. Front. Comput. Neurosci. 24:146. 10.3389/fncom.2010.00146 21152255 PMC2998859

[B44] Ratcliff R. (1978). A theory of memory retrieval. Psychol. Rev. 85, 59–108 10.1037/0033-295X.85.2.59

[B45] Rorie A. E. Gao J. McClelland J. L. Newsome W. T. (2010). Integration of sensory and reward information during perceptual decision-making in lateral intraparietal cortex (LIP) of the macaque monkey. PLoS ONE 5:e9308 10.1371/journal.pone.0009308 20174574 PMC2824817

[B46] Schultz W. (1998). Predictive reward signal of dopamine neurons. J. Neurophysiol. 80, 1–27. 9658025 10.1152/jn.1998.80.1.1

[B47] Serences J. T. (2008). Value-based modulations in human visual cortex. Neuron 60, 1169–1181. 10.1016/j.neuron.2008.10.051 19109919 PMC3384552

[B48] Shadlen M. N. Newsome W. T. (2001). Neural basis of a perceptual decision in the parietal cortex (area LIP) of the rhesus monkey. J. Neurophysiol. 86, 1916–1936 Available online at: http://jn.physiology.org/content/86/4/1916.full-text.pdf+html 11600651 10.1152/jn.2001.86.4.1916

[B49] Simen P. Contreras D. Buck C. Hu P. Holmes P. Cohen J. D. (2009). Reward rate optimization in two-alternative decision making: empirical tests of theoretical predictions. J. Exp. Psychol. 35, 1865–1897 10.1037/a0016926 PMC2791916 19968441

[B50] Summerfield C. Egner T. Mangels J. Hirsch J. (2006). Mistaking a house for a face: neural correlates of misperception in healthy humans. Cereb. Cortex 16, 500–508. 10.1093/cercor/bhi129 16014866

[B51] Summerfield C. Koechlin E. (2010). Economic value biases uncertain perceptual choices in the parietal and prefrontal cortices. Front. Hum. Neurosci. 4:208. 10.3389/fnhum.2010.00208 21267421 PMC3024559

[B52] Summerfield C. Tsetsos K. (2012). Building bridges between perceptual and economic decision-making: neural and computational mechanisms. Front. Hum. Neurosci. 6:70. 10.3389/fnins.2012.00070 22654730 PMC3359443

[B53] Sutton R. S. Barto A. G. (1998). Reinforcement Learning: Introduction. Cambridge, MA: MIT press.

[B54] Tom S. M. Fox C. R. Trepel C. Poldrack R. A. (2007). The neural basis of loss aversion in decision-making under risk. Science 315, 515–518. 10.1126/science.1134239 17255512

[B55] Watkins C. J. C. H. Dayan P. (1992). Q-Learning. Mach. Learn. 8, 279–292 10.1007/BF00992698

[B56] White C. N. Poldrack R. A. (2014). Decomposing bias in different types of simple decisions. J. Exp. Psychol. 40, 385–398. 10.1037/a0034851 24245536

[B57] Whiteley L. Sahani M. (2008). Implicit knowledge of visual uncertainty guides decisions with asymmetric outcomes. J. Vis. 8, 2.1–215. 10.1167/8.3.2 18484808 PMC2515365

[B58] Worsley K. J. (2001). Statistical analysis of activation images, in Functional MRI: an Introduction to Methods, eds Jezzard P. Matthews P. M. Smith S. M. (New York, NY: Oxford University Press), 251–270.

[B59] Wunderlich K. Rangel A. O'Doherty J. P. (2009). Neural computations underlying action-based decision making in the human brain. Proc. Natl. Acad. Sci. U.S.A. 106, 17199–17204. 10.1073/pnas.0901077106 19805082 PMC2761331

[B60] Yeung N. Nystorm L. E. Aronson J. A. Cohen J. D. (2006). Between-task competition and cognitive control in task switching. J. Neurosci. 26, 1429–1438. 10.1523/JNEUROSCI.3109-05.2006 16452666 PMC6675498

